# Supplementary figures and images for: Potential of mesenchymal- and cardiac progenitor cells for therapeutic targeting of B-cells and antibody responses in end-stage heart failure
Source: PLoS One. 2019 Dec 31;14(12):e0227283. doi: 10.1371/journal.pone.0227283 (PMC6938331; doi:10.1371/journal.pone.0227283)

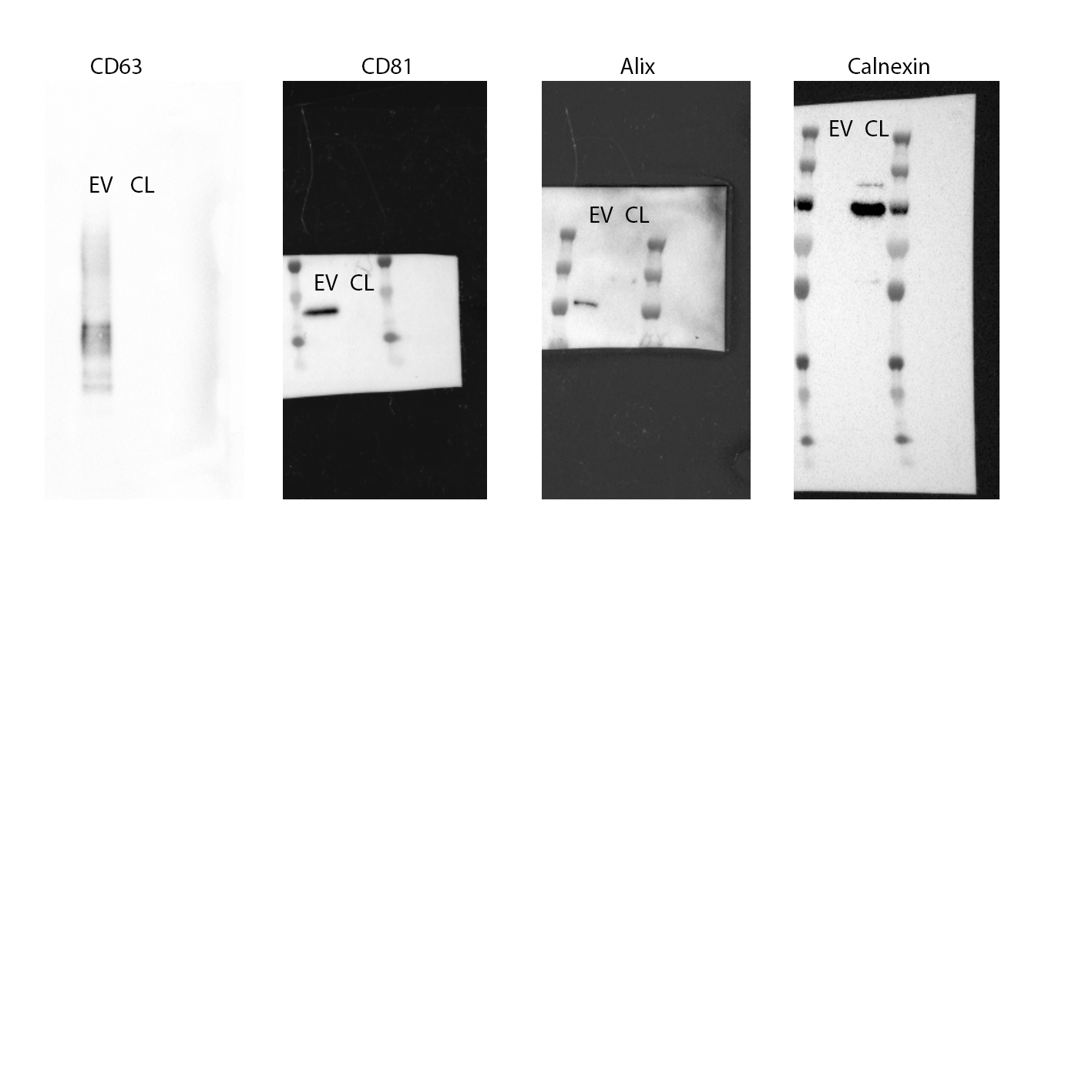

Supplement: S1 Fig — Proper isolation of extracellular vesicles (EV) was determined by the presence of CD63, CD81 and Alix and absence of the cellular marker Calnexin by Western Blotting. Cell lysates (Cl) were used as controls. (TIF) [file pone.0227283.s001.tif]
